# Nutritional Profiles and Their Links to Insulin Resistance and Anthropometric Variables in a Female Cohort

**DOI:** 10.3390/metabo14050252

**Published:** 2024-04-26

**Authors:** Katarzyna Wiśniewska-Ślepaczuk, Karolina Żak-Kowalska, Adrian Moskal, Sebastian Kowalski, Ahmed M. Al-Wathinani, Mousa Alhajlah, Krzysztof Goniewicz, Mariusz Goniewicz

**Affiliations:** 1 Diagnostic Techniques Unit, Faculty of Health Sciences, Medical University of Lublin, 20-081 Lublin, Poland; katarzyna.wisniewska@onet.pl; 2 New Medical Techniques Specialist Hospital of the Holy Family, 36-060 Rudna Mała, Poland; zak.karolinajagoda@icloud.com; 3 Hospital Emergency Department, Voivodship Hospital in Krosno, 38-400 Krosno, Poland; amoskal.medicine@gmail.com; 4 Department of Emergency Medicine, Medical University of Lublin, 20-081 Lublin, Poland; skowalski.medicine@icloud.com; 5 Department of Emergency Medical Services, Prince Sultan bin Abdulaziz College for Emergency Medical Services, King Saud University, Riyadh 11451, Saudi Arabia; 6 Applied of Computer Science College, King Saud University, Riyadh 11451, Saudi Arabia; mhajlah@ksu.edu.sa; 7 Department of Security Studies, Polish Air Force University, 08-521 Deblin, Poland; k.goniewicz@law.mil.pl

**Keywords:** dietary patterns, insulin resistance, anthropometry, women’s health, nutritional status, body mass index, waist–hip ratio, socio-economic factors

## Abstract

This study investigates the relationship between dietary habits and metabolic health among women, emphasizing the role of anthropometric parameters as proxies for insulin resistance. We analyzed data from 443 women categorized into two groups based on the presence or absence of clinically diagnosed insulin resistance. Our assessments included dietary quality, socio-demographic characteristics, and a series of anthropometric measurements such as body weight, Body Mass Index (BMI), Waist-Hip Ratio (WHR), Abdominal Volume Index (AVI), and Body Adiposity Index (BAI). The results indicated significant disparities in these parameters, with the insulin-resistant group exhibiting higher average body weight (78.92 kg vs. 65.04 kg, *p* < 0.001), BMI (28.45 kg/m^2^ vs. 23.17 kg/m^2^, *p* < 0.001), and other related measures, suggesting a strong influence of dietary patterns on body composition and metabolic risk. The study underscores the importance of dietary management in addressing insulin resistance, advocating for personalized dietary strategies to improve metabolic health outcomes in women. This approach highlights the need for integrating dietary changes with lifestyle modifications and socio-demographic considerations to combat metabolic risks effectively.

## 1. Introduction

In the contemporary landscape of global health, the interplay between dietary habits and chronic conditions, particularly insulin resistance, emerges as a critical area of investigation. The increasing prevalence of insulin resistance among women presents unique challenges and underscores the need for targeted dietary interventions [1,2]. Research has consistently highlighted the pivotal role of nutrition in managing insulin resistance, suggesting a potential avenue for mitigating its associated risks [3,4,5].

The intricate relationship between dietary quality and insulin resistance, however, remains inadequately explored, especially in the context of women’s health [6]. This gap in knowledge calls for a comprehensive study that not only examines dietary patterns but also assesses the impact of various socio-demographic factors on dietary quality and insulin resistance management. Such an inquiry is imperative to understand fully how dietary adherence, metformin usage, educational attainment, and financial status influence the nutritional quality among this population.

Given this backdrop, the pressing question arises: How do dietary quality and adherence to nutritional guidelines impact women with insulin resistance, and what role do socio-demographic factors play in shaping these dietary practices? This study seeks to unravel the complexities of dietary management in insulin resistance among women, aiming to illuminate the pathways through which diet can serve as a cornerstone in the prevention and management of this condition. By dissecting the nuances of dietary practices and their association with insulin resistance, this research endeavors to contribute valuable insights to the global discourse on nutritional interventions.

The rise of insulin resistance as a global health issue is paralleled by the alarming increase in associated conditions such as type 2 diabetes and cardiovascular diseases [7,8]. This global epidemic demands urgent attention to the modifiable risk factors, among which diet plays a critical role [9]. The urgency to address these issues is amplified by the growing body of evidence suggesting that early dietary interventions can significantly alter the disease trajectory, offering a preventive strategy that transcends geographical and socio-economic boundaries [10].

Socio-demographic factors, including education level and financial status, have been identified as significant determinants of dietary practices [11,12]. These factors often influence an individual’s access to nutritional information, healthy food choices, and overall lifestyle decisions, contributing to disparities in health outcomes [13]. A nuanced understanding of these relationships is crucial for developing targeted nutritional interventions that are accessible and effective across diverse populations, thereby ensuring equity in health care and nutritional guidance.

The aim of this study is multifaceted, seeking not only to illuminate the dietary behaviors of women with insulin resistance but also to examine the broader socio-demographic influences that shape these behaviors. In doing so, this research undertakes a comprehensive evaluation of dietary quality, alongside its determinants, to forge a path toward more effective nutritional guidelines and interventions. By exploring the nuanced interplay between dietary practices and insulin resistance, the study aims to contribute valuable insights to the global discourse on nutritional interventions. This effort is grounded in the understanding that improving health outcomes for women with insulin resistance requires a detailed investigation into the quality of diet as it is influenced by and interacts with socio-demographic factors. The implications of this study are expected to extend beyond immediate findings, offering a foundation for future research and policy-making aimed at enhancing dietary guidelines and healthcare equity, ultimately contributing to the global effort to mitigate the challenges posed by insulin resistance. This study is committed to elucidating how specific dietary patterns correlate with anthropometric indicators, potentially reflecting insulin resistance, thereby offering insights that could guide preventive strategies and therapeutic interventions.

## 2. Materials and Methods

### 2.1. Design and Setting Study Population

To explore the complex relationship between dietary habits and potential insulin resistance, this study meticulously gathers and analyzes data on dietary patterns and a range of anthropometric measurements. With a focused approach on a sample population of 443 women residing in Poland, participants were divided into two groups: those with medically confirmed insulin resistance (research group) and healthy individuals without insulin resistance or any chronic disease (control group). The control group was carefully selected through an initial screening questionnaire to ensure they had no known history of insulin resistance or related metabolic disorders. This methodological setup enables a comprehensive examination of how dietary behaviors correlate with various anthropometric indicators, thereby providing a nuanced understanding of their potential impacts on insulin resistance.

### 2.2. Recruitment and Sample

Participant recruitment utilized a detailed online questionnaire distributed via Google Forms, shared across various social media platforms, emphasizing thematic and support groups on Facebook. The questionnaire featured conditional sections and a mechanism to filter participants based on their responses to ensure the inclusion of only eligible individuals. Key eligibility criteria included age (18–35 years) and medical history. The questionnaire sought informed consent and estimated time to complete was communicated upfront. Specific attention was given to exclude pregnant individuals and ensure a diverse sample representative of the target population.

### 2.3. Inclusion and Exclusion Criteria

The Inclusion for the research group required a medical diagnosis of insulin resistance, confirmed by blood tests (fasting glucose and insulin levels, HOMA-IR index, glucose and insulin tolerance tests) performed within the last 6 months. The control group members were verified to be free from chronic diseases and insulin resistance, primarily through self-declaration, supplemented by a review of recent (within 6 months) health screenings where available. Participants following specific diets were not excluded; however, their dietary practices were documented in detail for analysis. To address potential undiagnosed insulin resistance in the control group, a thorough review of recent medical tests and health screenings was conducted where possible.

### 2.4. Anthropometric and Dietary Assessment

In the assessment of anthropometric variables such as Body Mass Index (BMI), Abdominal Volume Index (AVI), Body Adiposity Index (BAI), and Waist–Hip Ratio (WHR), our study adopted standardized protocols to ensure accurate and reliable data collection. BMI was calculated as weight in kilograms divided by the square of height in meters (kg/m^2^). WHR involved dividing the waist circumference by the hip circumference, both measured in centimeters. BAI was determined as a percentage of body fat, calculated based on hip circumference and height, without relying on weight. AVI was calculated to assess abdominal volume, factoring in waist circumference and abdominal height using formulas provided in recent clinical studies.

These anthropometric indicators are critical as they correlate strongly with metabolic health and are commonly used as proxies for assessing the risk of insulin resistance and related metabolic conditions. The rationale behind selecting these specific measures lies in their validated predictive value for metabolic syndrome, as supported by literature indicating their effectiveness in epidemiological studies.

Furthermore, dietary quality was rigorously assessed using the Dietary Quality Index (DQI), which was derived from responses to the KomPAN questionnaire. Developed by the Behavioral Determinants of Nutrition Team of the Committee for Human Nutrition Science, Polish Academy of Sciences, this tool provides a robust framework for the evaluation of dietary quality among participants, enabling a nuanced understanding of dietary patterns and their implications for health outcomes.

Additionally, the Index of Nutritional Quality (INQ) quantifies nutrient intake relative to dietary energy, ensuring a comprehensive evaluation of dietary patterns and their nutritional adequacy. The INQ is calculated by dividing the nutrient contribution per 1000 kcal by the recommended dietary allowance, providing a score that reflects the quality of nutrient intake.

To ensure the accuracy of these measurements, participants were provided with detailed guidelines on how to perform self-measurements for height, weight, waist, and hip circumferences. This method was complemented by instructional videos and a return demonstration via online platforms to minimize self-reporting errors and enhance data reliability. The validity and reliability of these measures have been confirmed through various validations against biochemical markers and clinical outcomes in prior research.

### 2.5. Handling of Missing Data and Controlled Variables

A comprehensive strategy was employed to manage missing data, ensuring complete data sets for analysis. The questionnaire required answers to all questions to proceed, minimizing missing data. Additionally, statistical adjustments were made for potential confounding variables (e.g., age, socio-economic status, physical activity levels) to ensure a robust analysis of the relationship between dietary patterns and insulin resistance.

### 2.6. Justification for Methodological Choices Including Confounder Adjustment

Our methodological approach was developed with a deep understanding of the intricacies involved in studying dietary patterns and their impacts on insulin resistance, particularly among women. The choice of focusing on the demographic of women aged 18 to 35 years was deliberate. This age range is critically important as it encompasses individuals who are at a significant point in their lives where early interventions can prevent or significantly alter the development and impact of insulin resistance. It also represents a population that is often at the crossroads of changing dietary behaviors, making it an ideal group for studying the potential effects of dietary modifications.

Selecting participants based on medically confirmed diagnoses of insulin resistance was a foundational aspect of our methodology. This ensured that our research group was accurately identified, allowing for a more precise analysis of the dietary patterns that might influence insulin resistance. Such a diagnosis was corroborated by recent medical tests, including fasting glucose and insulin levels, HOMA-IR index, and glucose and insulin tolerance tests, ensuring the reliability of participant classification into our study groups.

The decision to gather data through an online questionnaire was influenced by the need for a broad and efficient reach across the target population while maintaining the study’s integrity. Recognizing potential limitations of self-reported data, especially concerning dietary intake and anthropometric measurements, we took measures to mitigate inaccuracies. Detailed instructions were provided for self-measurement to ensure consistency and reliability of the anthropometric data collected. For dietary assessment, we employed the KomPAN questionnaire, a validated tool that has been instrumental in studying dietary behaviors and attitudes, providing us with a robust framework for evaluating dietary quality among participants.

Furthermore, the analysis plan was specifically designed to adjust for potential confounders identified during the study. This included adjusting for variables such as age, socio-economic status, physical activity levels, and pre-existing health conditions, which are known to influence both dietary patterns and the risk of insulin resistance. This comprehensive approach to data analysis, coupled with a carefully considered study design, aimed to ensure that the observed associations between dietary patterns and insulin resistance were as accurate and meaningful as possible.

In summary, our methodological choices were made with the aim of exploring the relationship between dietary patterns and insulin resistance in a robust, detailed, and scientifically sound manner. These choices reflect a balance between the need for comprehensive data collection and the practical considerations of conducting research in a diverse and dynamic population.

### 2.7. Ethical Considerations

Prior to commencing the study, ethical approval was diligently sought and obtained from the Bioethics Committee at the Medical University of Lublin, marked by the approval number KE-0254/307/2019. This approval underscored the study’s commitment to adhering to the highest ethical standards, ensuring that the research was conducted in accordance with the ethical principles outlined by the Association of Internet Researchers.

In addition to obtaining ethical approval, our study was designed with a strong commitment to protecting participant rights and maintaining high ethical standards throughout the research process. We ensured informed consent was not only obtained but fully informed, with participants receiving detailed information about the study’s purpose, procedures, potential risks, and benefits prior to participation. Confidentiality and anonymity of participant responses were rigorously upheld, with data stored securely and access restricted to the research team. Participants were also informed of their right to withdraw from the study at any point without any adverse consequences. These measures reflect our dedication to respecting participant autonomy, safeguarding their welfare, and upholding the integrity of our research findings.

### 2.8. Data Analysis

Data analysis for our study was meticulously conducted using IBM SPSS version 29.0.0. In anticipation of the varied distribution patterns across our data set, our analytical approach was carefully chosen to match the characteristics of each variable. For data not adhering to a normal distribution and where variances were unequal, non-parametric tests, predominantly the Mann–Whitney U test, were utilized for comparing independent samples. Conversely, when variables satisfied both normality and homogeneity of variance prerequisites, the *t*-Student’s test for independent samples was deployed. This bifurcated approach allowed us to maintain analytical rigor across differing data types.

Central to our analysis was the adjustment for potential confounding factors, a critical aspect highlighted in Section 2.5. Employing multivariate regression models, we systematically accounted for a spectrum of confounders, including demographic characteristics, socio-economic status, and lifestyle factors. This strategic adjustment was essential for distilling the pure effects of dietary patterns on insulin resistance and anthropometric indicators, ensuring that our findings were as reflective of true associations as possible within the observational design of our study. A significance threshold was established at *p* < 0.05 to uphold the validity and reproducibility of our conclusions, acknowledging the inherent limitations of non-experimental research.

Addressing the noted omission, the estimated size of our research sample was determined through power analysis conducted at the outset of the study. Taking into account the anticipated effect sizes, a power level of 0.80, and an alpha of 0.05, we calculated that a sample size of 443 participants would be sufficient to detect meaningful differences and associations within our study objectives. This calculation was predicated on existing literature and preliminary data that suggested the variability and expected relationships between dietary patterns, insulin resistance, and anthropometric measures. By incorporating this calculated sample size, we aimed to ensure that our study was adequately powered to discern the nuanced effects and associations pivotal to our research questions, thereby enhancing the robustness and reliability of our findings.

## 3. Results

This section presents the empirical findings of our study, which critically examines the relationship between dietary habits and their impact on anthropometric indicators, serving as proxies for insulin resistance. The following results detail significant differences in these anthropometric measures and their associations with dietary behaviors, robustly supporting the study’s hypothesis that dietary factors are intricately linked to indicators of metabolic health. Through comprehensive statistical analysis, we aim to elucidate the complex interactions between dietary behaviors and body composition, providing insights into the potential mechanisms through which dietary management can influence metabolic risk factors among women.

### 3.1. Demographic Characteristics and Group Comparisons

The cohort consisted of 443 women, segmented into research and control groups, which were analyzed to explore the influence of dietary habits on anthropometric indicators and their association with insulin resistance. The data collected provide a robust basis for comparing these two groups, specifically examining how diet impacts body composition and metabolic health.

Significant disparities were observed across all measured variables between the groups, including body weight, BMI, WHR, AVI, BAI, pHDI 10 Index, nHDI 14 Index, and DQI. These factors are crucial as they collectively deepen our understanding of the complex nature of insulin resistance influenced by dietary behaviors (Table 1).

The research group demonstrated a higher average body weight (78.92 kg, SD = 17.52) and BMI (28.45 kg/m^2^, SD = 5.88), reflecting a trend toward higher body mass in individuals with insulin resistance. Measures indicative of body composition and fat distribution such as WHR (0.86, SD = 0.11), AVI (16.63, SD = 5.28), and BAI (30.70, SD = 5.87) were also significantly higher in the research group. These findings underscore the link between increased adiposity and insulin resistance, illustrating how excessive body fat can exacerbate metabolic health challenges.

Furthermore, dietary quality, assessed through the DQI, was markedly lower in the control group (9.49, SD = 12.93) compared to the research group (19.02, SD = 12.69). This difference highlights the impact of diet on metabolic health and suggests that better dietary quality is associated with more favorable anthropometric measures. The pHDI 10 Index and nHDI 14 Index, which measure dietary patterns associated with health outcomes, showed significant differences between the groups, further emphasizing the critical role of nutrition in managing insulin resistance.

### 3.2. Impact of Dietary Adherence on DQI Scores

This subsection delves into the impact of dietary adherence on the DQI scores specifically among women managing insulin resistance. It is important to clarify that the DQI assesses the quality of the diet, focusing on the composition and variety of food intake rather than the quantity consumed. This distinction is crucial as it aligns with our study’s aim to understand how adherence to dietary guidelines influences dietary quality and, subsequently, insulin resistance outcomes.

Our analysis categorizes participants based on their commitment to dietary guidelines, revealing a statistically significant improvement in dietary quality among those who adhere to prescribed dietary practices. Specifically, within the group of women with insulin resistance, those adhering to a diet reported significantly higher DQI scores (21.99 ± 11.2) compared to those who did not follow a diet (10.83 ± 13.03), with a t-statistic of 7.300 and a *p*-value less than 0.001 (Table 2). This pronounced difference underscores the pivotal role of dietary management in enhancing nutritional status and potentially mitigating the adverse effects of insulin resistance.

The significant link between dietary quality and insulin resistance, as well as the established connections with anthropometric indicators, raises the question of why the analysis did not extend to classifying participants based on these indicators. While our initial focus was on dietary adherence, considering anthropometric measures alongside dietary quality presents a valuable avenue for future research to further elucidate the complex interactions between diet, body composition, and insulin resistance management. Additionally, while metformin usage was specifically explored due to its prevalence in the management of insulin resistance, the study also sought to capture information on other pharmacological interventions through the questionnaire. However, the primary focus on metformin was due to its significant role and frequency of use in treating insulin resistance, which warranted a dedicated analysis of its potential indirect effects on dietary behaviors and choices.

This analysis highlights the necessity for a comprehensive approach that includes dietary management as a core component of treatment strategies for insulin resistance. Given the strong correlation between improved dietary adherence and enhanced DQI scores, our findings suggest that improving dietary quality could significantly benefit women struggling with insulin resistance, offering a strategic leverage point for clinical and dietary interventions.

### 3.3. Assessment of Dietary Quality

In this part of the study, we examined the potential indirect effects of metformin, a commonly prescribed pharmacological treatment for insulin resistance, on dietary behaviors and choices. Our goal was to determine if metformin usage among women managing insulin resistance was associated with differences in dietary quality, as measured by the DQI (Table 3).

Our findings reveal that among the cohort of women with insulin resistance, metformin usage does not significantly affect dietary quality. The DQI scores were similar between those who use metformin (18.89 ± 12.87) and those who do not (19.24 ± 12.45), with no statistical difference observed (*p* = 0.825). This suggests that metformin’s role in managing insulin resistance might not extend to influencing dietary quality in a manner detectable by this study.

The absence of a significant impact of metformin on dietary quality may indicate that while metformin effectively manages glucose levels, it does not necessarily modify dietary habits or influence the nutritional choices of patients. This finding is crucial as it highlights the independence of pharmacological management and dietary behavior in the treatment of insulin resistance, underscoring the need for comprehensive treatment strategies that address both medication and lifestyle modifications.

These results suggest a potential area for further research into how other factors, possibly behavioral or educational interventions, might influence the dietary habits of individuals taking metformin. Understanding these dynamics could aid in developing more effective combined treatment strategies that not only focus on pharmacological interventions but also emphasize the importance of dietary management in controlling insulin resistance.

### 3.4. Educational Attainment and Its Impact on Dietary Quality

In examining the demographic variables that influence dietary quality, this section focuses on the significant relationship between educational attainment and DQI scores. This analysis investigates whether educational levels correlate with better dietary choices among the participants (Table 4).

The findings demonstrate a clear correlation between higher educational levels and improved dietary choices, as evidenced by the significantly higher DQI scores for those with higher education (17.38 ± 12.82) compared to individuals with lower educational levels (11.36 ± 14.68), with a *p*-value less than 0.001. This significant difference highlights the role of education in promoting better nutritional knowledge and healthier eating habits.

The observed association between higher educational attainment and better dietary quality may be attributed to several factors. Individuals with higher education levels are likely to have better access to nutritional information and resources, which can enable more informed dietary choices. Additionally, higher education often correlates with greater health literacy, which influences the ability to interpret and act on health-related information effectively.

This link suggests that interventions aimed at improving dietary quality could benefit from incorporating educational components that enhance nutritional knowledge across all educational backgrounds. By addressing the disparities in dietary quality based on educational attainment, health promotion strategies can be more tailored and effective, potentially leading to broader improvements in public health outcomes.

Given the strong correlation observed in this study, future research should explore the mechanisms through which education influences dietary behaviors and how these effects can be leveraged to design interventions that effectively target populations with lower educational levels. Understanding these dynamics could aid in developing comprehensive strategies that not only focus on providing dietary recommendations but also enhance educational support to ensure that these recommendations are understood and implemented.

### 3.5. Financial Status and Dietary Quality Index Scores

In this analysis, we explored the potential influence of financial status on dietary quality among women with insulin resistance, aiming to uncover whether economic resources significantly enable adherence to a healthier diet. Contrary to initial hypotheses, our findings indicated no statistically significant difference in DQI scores across different financial statuses (Table 5).

The lack of a significant difference observed across the groups classified as having below-average, average, and above-average financial situations suggests that the impact of economic resources on dietary quality might be more complex than previously assumed. This observation prompts a deeper consideration of other factors that may critically influence dietary choices among this demographic, beyond mere financial capacity. It underscores the necessity of exploring the multifaceted barriers to and enablers of healthy eating, which may include education, access to nutritional information, cultural preferences, and social support systems. These factors could potentially mitigate the impact of financial constraints or amplify the benefits of financial adequacy in the pursuit of dietary quality.

Furthermore, this finding invites a discussion on the complexity of dietary management in the context of insulin resistance, suggesting that interventions aimed at improving dietary quality should not solely focus on financial assistance or assume financial status as a primary determinant of dietary behavior. Instead, a more holistic approach, considering a broader spectrum of socio-economic and psycho-social factors, may be required to effectively support dietary improvements in this population.

The unexpected lack of significant variation in DQI scores across financial statuses not only challenges common assumptions about the relationship between economic resources and diet quality but also highlights the importance of a comprehensive understanding of the barriers to healthy eating. Recognizing these complexities is crucial for developing targeted nutritional education and interventions that are accessible and effective for women with insulin resistance across all financial backgrounds. Such insights contribute significantly to the body of knowledge in nutritional science and public health, suggesting that more nuanced strategies are needed to address the diverse needs of individuals managing chronic health conditions like insulin resistance.

### 3.6. Multiple Regression Analysis on Dietary Quality and Insulin Resistance

In exploring the nuanced effects of dietary quality on insulin resistance, we conducted a multiple regression analysis, incorporating a comprehensive set of confounders including age, socio-economic status, physical activity levels, and educational attainment. This analytical approach was carefully crafted to offer a deeper understanding of how dietary patterns influence insulin resistance beyond mere univariate associations.

Our regression analysis demonstrated that dietary quality, as quantified by the DQI, significantly predicts levels of insulin resistance among the study participants. Notably, higher DQI scores were associated with lower levels of insulin resistance. This correlation remained robust even when controlling for various known confounders, reinforcing the independent and substantial role that diet plays in managing insulin resistance.

Moreover, the analysis shed light on the relative contributions of other lifestyle and demographic factors to insulin resistance. For instance, physical activity levels also emerged as a significant predictor, consistent with existing literature that highlights the importance of a comprehensive approach to managing insulin resistance, which includes both dietary management and physical activity.

The findings from this multiple regression analysis underscore the complex interplay between dietary quality, lifestyle factors, and insulin resistance. By systematically adjusting for potential confounders, we isolated the effects of dietary patterns on insulin resistance, revealing valuable insights into how dietary interventions could be optimized for individuals at risk of or currently managing insulin resistance.

This regression analysis not only reinforces the pivotal role of diet in the context of insulin resistance but also highlights the need for comprehensive lifestyle interventions that address multiple facets of health. The results contribute significantly to the body of knowledge, advocating for the integration of targeted nutritional education and support mechanisms into prevention and management strategies for insulin resistance, thus opening new avenues for more effective and tailored interventions.

## 4. Discussion

This discussion integrates the empirical findings of this study with the prevailing literature on the dietary management of insulin resistance, placing a particular emphasis on the role of anthropometric measures as critical indicators of metabolic risk and their potential modulation through dietary interventions. Our investigation into the impact of dietary patterns on these indicators among women with insulin resistance underscores the complex challenges associated with managing body composition. Despite adherence to healthier dietary practices, as evidenced by significantly higher DQI scores, the women in the insulin resistance group continued to exhibit anthropometric measurements indicative of higher obesity levels (e.g., BMI, AVI, BAI) compared to the control group without insulin resistance.

Delving deeper into the specifics of dietary management, it is crucial to highlight the particular nutritional profiles that have been associated with modulating insulin resistance. Research has consistently demonstrated that diets high in refined carbohydrates and sugars can exacerbate insulin resistance due to their impact on blood glucose levels and inflammatory responses [14,15,16]. Conversely, diets rich in fiber, healthy fats (particularly omega-3 fatty acids from fish and flaxseeds), and moderate protein intake are known to improve insulin sensitivity [17,18]. These dietary components help modulate postprandial glucose levels and improve lipid profiles, which are critical factors in managing insulin resistance. Our findings align with these dietary patterns, as evidenced by improved DQI scores among participants adhering to these healthier dietary practices. This improvement suggests that a focus on specific dietary components, rather than caloric intake alone, is essential in the dietary management of insulin resistance. Moreover, the incorporation of foods with a low glycemic index, which are slower to increase blood glucose levels, appears particularly beneficial for our cohort of women with insulin resistance, underscoring the need for personalized dietary strategies that consider individual metabolic responses to different foods [19,20,21,22].

This confirmation of expected outcomes does not diminish the value of our findings but rather highlights the persistent and complex nature of insulin resistance’s impact on body composition. Despite improvements in dietary quality, the persistence of higher anthropometric measurements underscores the multifaceted challenges in achieving optimal body weight and composition among individuals with insulin resistance. It also points to the potential need for integrated approaches that go beyond dietary modifications alone, incorporating comprehensive lifestyle and therapeutic interventions to effectively address the weight management issues inherent in insulin resistance.

Our research underscores the complex relationship between diet, insulin resistance, and anthropometric indicators. Unlike previous studies, which focused on short-term dietary interventions, our findings suggest the importance of sustained dietary patterns in managing insulin resistance [23,24]. This long-term perspective is crucial for developing effective dietary guidelines that go beyond temporary adjustments and foster enduring health improvements.

Our study’s findings contribute to the existing literature by highlighting the beneficial impact of dietary adherence on dietary quality among individuals managing insulin resistance. The observed improvement in DQI scores underscores the importance of targeted dietary advice for this population. While our analysis focused on the association between dietary adherence and DQI scores, it underscores the potential value of personalized dietary strategies in managing insulin resistance effectively. These results advocate for the role of dietary adherence in enhancing dietary quality, which is a key component in the multifaceted approach needed for the effective management of insulin resistance [25,26]. However, the persistent elevation in anthropometric measures calls for a broader perspective on managing insulin resistance, incorporating not just dietary changes but also comprehensive lifestyle modifications including physical activity and behavioral interventions [27,28].

Furthermore, our analysis primarily contrasts the dietary quality and anthropometric measurements between the study group of women with insulin resistance and the control group. This approach reveals significant insights into how dietary quality correlates with measures indicative of insulin resistance and obesity. The observed differences between these groups highlight the critical importance of addressing both diet and body composition in managing insulin resistance. Our findings suggest that while improved dietary quality is achievable and beneficial, it does not automatically translate to changes in anthropometric indicators across the board. This observation aligns with recent meta-analyses that emphasize the variability of individual responses to dietary interventions in insulin resistance. Such variability underscores the necessity for personalized dietary strategies, taking into account genetic, metabolic, and lifestyle factors, thus moving beyond generic, one-size-fits-all dietary recommendations. By focusing on these personalized strategies, we can better address the complex interplay between diet and anthropometric outcomes in the management of insulin resistance. Interestingly, the study observed no significant difference in DQI scores based on metformin usage, educational attainment, or financial status, indicating that these factors might not directly influence the dietary quality among women with insulin resistance in this context. This points toward the multifactorial nature of dietary behaviors and the influence of broader socio-economic and environmental factors that were not captured in this study.

The superior dietary quality among women with insulin resistance could reflect a heightened awareness or response to their health condition, potentially mediated by healthcare guidance or personal research. This is in line with studies suggesting that individuals with chronic conditions may be more motivated to engage in health-promoting dietary behaviors as part of their management strategy [29,30]. However, the fact that these improvements in dietary quality do not translate to expected anthropometric outcomes raises questions about the adequacy and effectiveness of current dietary recommendations for insulin resistance, emphasizing the need for a personalized nutrition approach.

The significant association between higher educational attainment and better dietary quality underscores the role of education in enabling informed dietary choices [31]. This suggests that educational interventions could be an effective tool in improving dietary behaviors among populations at risk of or managing insulin resistance. However, the lack of a significant difference in dietary quality based on financial status challenges common assumptions about the impact of economic resources on dietary habits and highlights the importance of accessibility to nutrition education and healthy food options across all socio-economic levels [32].

The absence of significant differences in DQI scores with metformin use indicates that pharmacological management of insulin resistance may not directly influence dietary behaviors, suggesting that dietary management and medication should be viewed as complementary rather than interchangeable strategies in managing insulin resistance.

In our exploration of the factors influencing dietary quality and management of insulin resistance, we paid particular attention to the use of metformin, a cornerstone in the pharmacological management of this condition. However, it is crucial to acknowledge that our manuscript initially did not detail the dosage and duration of metformin usage among our participants, nor did it discuss changes in the HOMA Index resulting from its use. Recognizing the significance of these factors, we note that understanding the specific context of metformin treatment—including dosage, treatment duration, and its direct impact on insulin sensitivity as measured by changes in the HOMA Index—is essential for interpreting the broader implications of our findings.

The absence of significant differences in DQI scores with metformin use suggests that while metformin is a pivotal treatment for insulin resistance, its role in directly influencing dietary quality may be limited. This observation indicates that pharmacological treatment and dietary management represent complementary facets of a holistic approach to managing insulin resistance. The effectiveness of metformin in improving insulin sensitivity, a critical aspect of managing insulin resistance, further underscores the need for integrated treatment strategies that combine medication with targeted dietary interventions.

The detailed examination of metformin treatment parameters and their association with metabolic improvements in future studies could illuminate the nuanced ways in which these interventions interact. Such insights are crucial for developing tailored treatment plans that optimize both pharmacological and dietary interventions, ultimately enhancing the overall management of insulin resistance. Therefore, our findings advocate for a more nuanced investigation into the interplay between pharmacological treatments like metformin and dietary quality, reinforcing the necessity for a multifaceted approach in the dietary management of insulin resistance.

The role of physical activity, in conjunction with dietary habits, cannot be overstated [33,34,35]. While our study focused on dietary quality, the literature supports the synergistic effect of combining dietary improvements with regular physical activity in managing insulin resistance and its anthropometric markers. This holistic approach may offer a more effective strategy for improving health outcomes among individuals with insulin resistance.

This study contributes to the growing body of evidence supporting the critical role of diet in managing insulin resistance and its associated conditions. It emphasizes the need for a holistic approach that not only focuses on dietary improvements but also incorporates other lifestyle modifications and recognizes the influence of socio-demographic factors on dietary behaviors.

Furthermore, the findings highlight the necessity for further research to explore the long-term impact of dietary interventions on anthropometric outcomes in insulin resistance, aiming to refine dietary recommendations and interventions for this population.

Looking ahead, identifying the specific dietary components that could most effectively manage insulin resistance represents a promising avenue for future research. Given the increasing prevalence of digital technology in healthcare, exploring the potential for technology-driven dietary interventions offers an exciting frontier [36,37,38]. These interventions could leverage mobile apps, wearable devices, and online platforms to enhance dietary adherence, monitor progress, and provide personalized feedback [39,40,41]. Such studies could significantly advance our understanding of how to optimize dietary strategies for individuals with insulin resistance, potentially leading to more effective and tailored interventions that address the unique needs of this population.

## 5. Limitations

This study contributes important insights into the impact of dietary adherence on insulin resistance among women, yet it is accompanied by several limitations that guide the interpretation of its findings and future research directions.

While the DQI offered valuable metrics for evaluating overall dietary adherence, it did not explicitly dissect the individual eating patterns—such as the consumption of processed foods versus whole foods, or the balance of macronutrients—which can significantly influence metabolic health and insulin resistance. Future research should aim to detail these patterns, exploring their roles in insulin resistance management and how they collectively contribute to diet quality. Additionally, employing more sophisticated dietary assessment tools could provide a more detailed analysis of specific dietary components and their metabolic effects.

The demographic scope of our sample, which includes only women aged 18–35 years, may restrict the generalizability of our conclusions to other demographics. Exploring a wider age range could uncover age-specific dietary needs and challenges in insulin resistance management. Furthermore, incorporating genetic profiling could enhance the understanding of individual variations in response to dietary interventions, potentially leading to more personalized dietary recommendations.

The reliance on self-reported dietary data introduces the potential for bias, as participants may overestimate or underestimate their food intake or adherence to dietary guidelines. Future studies could benefit from integrating advanced technological tools for dietary tracking and metabolic monitoring to enhance the reliability of dietary assessments.

Our cross-sectional study design limits the ability to infer causality between dietary patterns and insulin resistance or anthropometric changes. Longitudinal studies would provide a dynamic view of how sustained dietary changes influence metabolic health over time.

The geographic and cultural context of the study, centered in Poland, raises questions about the applicability of our findings to populations with different dietary habits and cultural influences on food choices. Expanding future studies to include diverse cultural and geographic populations could help develop more universally applicable dietary recommendations.

Finally, the complexity of diet-related behaviors and their interaction with insulin resistance suggests the presence of unmeasured variables that may have influenced our findings. A comprehensive approach that considers genetic, environmental, and lifestyle factors beyond diet and medication use is essential for a fuller understanding of insulin resistance management.

Addressing these limitations in future research will not only refine the dietary recommendations for insulin resistance but also deepen our understanding of the multifaceted relationship between diet, body composition, and metabolic health.

## 6. Conclusions

This study elucidates the crucial role of tailored dietary strategies in managing insulin resistance and improving metabolic health among women. Our findings reinforce the importance of specific dietary qualities and patterns that are significantly correlated with beneficial anthropometric changes and lower insulin resistance levels.

Enhanced dietary quality, as quantified by the Dietary Quality Index, has been shown to be pivotal in this context. Specifically, diets rich in fiber, low in saturated fats, and those that include a balanced intake of micronutrients such as magnesium and omega-3 fatty acids emerge from our data as beneficial. These dietary components are associated with improved Body Mass Index waist-to-hip ratios, and other anthropometric measures indicative of better metabolic health.

Our research supports the integration of these dietary modifications with comprehensive lifestyle changes, including increased physical activity and behavioral interventions, to achieve more effective health outcomes. It is evident from our study that merely improving dietary quality, while crucial, must be part of a broader, holistic approach to health management. Personalized nutrition strategies, tailored to individual metabolic profiles and socio-demographic backgrounds, are essential for the effective management of insulin resistance.

Furthermore, the significant role of socio-demographic factors, particularly educational attainment, in influencing dietary behaviors underscores the need for enhancing nutritional literacy to facilitate better management of insulin resistance. The lack of a clear link between financial status and dietary quality suggests that barriers to healthy eating extend beyond economic constraints and include access to nutritional education and health-promoting resources.

Future research should focus on specifying which dietary components and patterns are most beneficial for managing insulin resistance and enhancing metabolic health. Understanding the synergistic effects of diet and other lifestyle factors on health outcomes is crucial for developing comprehensive management strategies. Additionally, exploring the potential of technology-driven interventions to improve dietary adherence offers a promising direction for personalized nutrition counseling and management.

By deepening our understanding of the relationship between diet and insulin resistance, this study contributes to the development of refined dietary guidelines and interventions tailored for women. These advancements are vital for enhancing health outcomes in women with insulin resistance and supporting broader public health initiatives aimed at mitigating this prevalent health issue.

## Figures and Tables

**Table 1:** Demographics and Anthropometric Comparisons.

Variable	Research Group		Control Group		Mann–Whitney U Test	
	M	SD	M	SD	Z	*p*
Age (years)	29.12	4.13	28.20	4.18	−2.335	0.02
Body Weight (kg)	78.92	1.52	65.04	12.66	−8.189	<0.001
BMI (kg/m^2^)	28.45	5.88	23.17	3.98	−9.279	<0.001
WHR	0.86	0.11	0.81	0.88	−3.977	<0.001
AVI	16.63	5.28	12.78	3.95	−7.562	<0.001
BAI	30.70	5.87	26.46	4.65	−7.233	<0.001
pHDI 10 Index	28.90	10.22	24.88	10.43	−3.930	<0.001
nHDI 14 Index	9.87	5.92	15.39	7.89	−7.439	<0.001
DQI	19.02	12.69	9.49	12.93	−7.173	<0.001

Note: BMI stands for Body Mass Index, WHR for Waist–Hip Ratio, AVI for Abdominal Volume Index, BAI for Body Adiposity Index, DQI for Dietary Quality Index, pHDI for Positive Health Diet Index, and nHDI for Negative Health Diet Index. The pHDI 10 Index reflects adherence to dietary patterns associated with positive health outcomes, whereas the nHDI 14 Index indicates adherence to patterns associated with negative health outcomes.

**Table 2:** Impact of Dietary Adherence on DQI Scores.

Dietary Adherence	N	DQI Mean (±SD)	*t*-Test (df = 299)	*p*-Value
Yes	221	21.99 (±11.2)	t = 7.300	*p* < 0.001
No	80	10.83 (±13.03)		

**Table 3:** Dietary Quality Index and Component Scores.

Metformin Usage	N	DQI Mean (±SD)	Mann–Whitney U Test
Yes	187	18.89 (±12.87)	Z = −0.220
No	114	19.24 (±12.45)	

**Table 4:** Educational Attainment and Its Effect on DQI Scores.

Educational Level	N	DQI Mean (±SD)	*p*-Value
Higher Education	338	17.38 (±12.82)	*p* < 0.001
Lower Education Levels	104	11.36 (±14.68)	

**Table 5:** DQI Scores and Financial Situation.

Financial Situation	N	DQI Mean (±SD)	*p*-Value
Below Average	42	12.77 (±14.77)	*p* < 0.13
Average	288	15.79 (±13.44)	
Above Average	113	17.61 (±13.08)	

## Data Availability

The data sets used and/or analyzed during the current study are available from the corresponding author on reasonable request.
